# Gulf War Illness, Fibromyalgia, Myalgic Encephalomyelitis/Chronic Fatigue Syndrome and Long COVID Overlap in Common Symptoms and Underlying Biological Mechanisms: Implications for Future Therapeutic Strategies

**DOI:** 10.3390/ijms26189044

**Published:** 2025-09-17

**Authors:** David Mantle, Joan Carles Domingo, Beatrice Alexandra Golomb, Jesús Castro-Marrero

**Affiliations:** 1 Pharma Nord (UK) Ltd., Morpeth NE61 2DB, Northumberland, UK; dmantle@pharmanord.com; 2 Department of Biochemistry and Molecular Biomedicine, Faculty of Biology, University of Barcelona, 08028 Barcelona, Spain; jcdomingo@ub.edu; 3 Department of Medicine, UC San Diego School of Medicine, La Jolla, CA 92093, USA; bgolomb@health.ucsd.edu; 4 Research Unit in Myalgic Encephalomyelitis/Chronic Fatigue Syndrome and Long COVID, Division of Rheumatology Research, Vall d’Hebron Hospital Research Institute, Universitat Autònoma de Barcelona, 08035 Barcelona, Spain

**Keywords:** Gulf War Illness, myalgic encephalomyelitis/chronic fatigue syndrome, fibromyalgia, long COVID, coenzyme Q10, mitochondrial dysfunction, oxidative stress, apoptosis/ferroptosis, neuroinflammation, immune dysregulation

## Abstract

Although Gulf War Illness (GWI), fibromyalgia (FM), myalgic encephalomyelitis/chronic fatigue syndrome (ME/CFS) and long COVID have distinct origins, in this article we have reviewed evidence that these disorders comprise a group of so-called low-energy associated disorders with overlapping common symptoms underlying pathology. In particular, evidence for mitochondrial dysfunction, oxidative stress, inflammation, immune dysregulation, neuroendocrine dysfunction, disrupted brain–gut-microbiome axis, apoptosis/ferroptosis and telomere shortening as common features in the pathogenesis of these disorders has been identified. Given the role of coenzyme Q10 (CoQ10) in promoting normal mitochondrial function, as an antioxidant, antiinflammatory and antiapoptotic and antiferroptotic agent, there is a rationale for supplementary CoQ10 in the management of these disorders. The reported benefits of supplementary CoQ10 administration in GWI, FM, ME/CFS and long COVID have been reviewed; the potential benefit of supplementary CoQ10 in reducing telomere shortening and improving the efficiency of stem cell transfer relevant has also been identified as promising therapeutic strategies in these disorders. This review advances beyond previous systematic reviews and consensus statements on overlapping similar symptoms and underlying biological pathomechanisms in these complex disorders.

## 1. Introduction

Gulf War Illness (GWI), fibromyalgia (FM), myalgic encephalomyelitis/chronic fatigue syndrome (ME/CFS), and long COVID are chronic, multifaceted, and poorly understood disabling disorders affecting millions of patients worldwide. The increasing prevalence of these disorders in the general population has been estimated as 2–6% for FM [1], 0.5–1% for ME/CFS [2], 6–10% for long COVID [3], predominantly more common in women than in men, with 25–35% of Gulf War veterans affected by GWI [4]. It should be noted that prevalence rates for these disorders vary somewhat between different epidemiological studies, and the figures quoted are estimates based on data from several sources.

These disorders share significant overlapping common symptoms, underlying biological pathomechanisms, and impacts on daily life, often resulting in similar management strategies [4,5,6]. These disorders present challenges in medical practice with regard to clinical diagnosis; whilst there are some specific diagnostic criteria for GWI (the Kansas case criteria, together with the less-restrictive Centers for Disease Control and Prevention (CDC) clinical definition), the diagnostic objectivity requires further improvement [7,8].

In this review we have compared what is known about the similarities and differences in shared symptoms but distinct origins and updated case criteria in GWI, FM, ME/CFS, and long COVID. Gulf War Illness, affecting veterans of the 1990–91 Gulf War, includes fatigue, pain, and cognitive issues linked to environmental exposure and genetic susceptibility [4]. Fibromyalgia, historically misunderstood, was formally recognized in 1990 with diagnostic criteria focusing on widespread pain and tender points, later revised to include fatigue and cognitive symptoms [9]. ME/CFS, dating back to the 19th century, gained prominence in the 1980s; its 2015 diagnostic criteria require post-exertional malaise, unrefreshing sleep, and cognitive or autonomic dysfunction [10]. Long COVID emerged in 2020 following acute SARS-CoV-2 infection, presenting with fatigue, brain fog, and multisystem symptoms lasting beyond four weeks, often resembling ME/CFS [11]. While all four disorders rely on clinical evaluation and symptom duration; their histories reflect evolving recognition, from initial skepticism to growing acceptance, with long COVID now driving research that may illuminate shared biological pathomechanisms across these neglected complex disorders.

In addition, there are no clear objective case criteria or specific biomarkers, nor any effective Food and Drug Administration (FDA)-approved treatments, with the exception of four drugs currently available for treating FM (duloxetine, pregabalin, milnacipran, and recently cyclobenzaprine). The above issues lead to variability in diagnosis, in monitoring illness severity, in predicting treatment response and in developing targeted therapies for these disorders, in turn highlighting a significant unmet medical need [4,5,6].

All of these disorders are characterized by common symptoms [4,11,12,13]; in particular persistent fatigue, post-exertional malaise (PEM) as a hallmark symptom that is not alleviated by rest, that often severely influences daily functioning. Other common symptoms include: (i) cognitive dysfunction (commonly referred to as *brain fog*), including problems with memory and concentration, and mental clarity, is prevalent in all four disorders; (ii) widespread musculoskeletal pain, defined as chronic pain especially in muscles and joints, is a hallmark symptom of FM and is commonly seen also in patients with GWI, ME/CFS and long COVID; (iii) sleep disturbance, including difficulty sleeping, unrefreshing sleep, and disrupted sleep patterns are seen in all these disorders; (iv) autonomic dysfunction occurs in many of these disorders, including dysregulation of the autonomic nervous system, leading to autonomic symptoms such as dizziness, light-headedness, orthostatic intolerance, and tachycardia; and (v) muscle weakness, particularly in FM, ME/CFS, and long COVID, is reported frequently, along with a general feeling of exhaustion that is disproportionate to physical and mental activity.

A growing body of literature has reported overlapping potential biological mechanisms in these disorders [14,15]. The pathophysiology of these disorders is still being explored, but recent research points to several shared biological mechanisms that may underlie the clinical symptoms. In terms of pathology, these disorders all show evidence of immune dysfunction, and at the cellular level there is evidence for an impaired mitochondrial function, increased oxidative stress, inflammation, apoptosis/ferroptosis and telomere shortening, as discussed in subsequent sections of this article [15,16]. Thus, GWI, FM, ME/CFS and long COVID comprise a group of so-called low-energy disorders with overlapping common symptoms and underlying pathophysiological mechanisms. Low-energy associated disorders are characterized by primary disabling fatigue that reduces the body’s capacity to perform daily activities, affecting both physical and mental functions.

Given the key role of CoQ10 in promoting normal mitochondrial function, as an antioxidant, antiinflammatory and antiapoptotic and antiferroptotic agent, in this article we have reviewed the potential role of supplemental CoQ10 in the therapeutic management of these disorders. A potential role for supplemental CoQ10 in reducing telomere shortening, and in improving the efficiency of stem cell therapy of relevance to these disorders has also been reviewed [17,18,19].

## 2. Common Symptoms in Low-Energy Associated Disorders

### 2.1. Fatigue and Post-Exertional Malaise

Debilitating fatigue and post-exertional malaise (PEM) are not the same, although they share some similarities. Debilitating fatigue is a broad term for extreme and severe tiredness that can be a symptom of many chronic illnesses, while PEM, also known as post-exertional symptom exacerbation (PESE) is a hallmark symptom of ME/CFS and sometimes GWI and long COVID, involving a worsening of symptoms following even minimal physical, and/or mental activity [6,20,21].

Post-exertional malaise can exacerbate a wide range of core symptoms, including fatigue, cognitive difficulties (*brain fog*), pain, sleep impairments and sensitivity to light and sound in these disorders. It can severely limit a person’s ability to participate in daily activities, making it difficult to work, attend school, or engage in social activities. The key is that the severity of symptoms is often disproportionate to the exertion that triggered it, and can be delayed, sometimes appearing 24–72 h after exertion, and may persist for months. Recovery from PEM can be slow and prolonged, potentially lasting for days, weeks, or even months [3,22].

Managing PEM often involves pacing, which is a strategy of balancing activity and rest to avoid triggering PEM episodes. PEM management is highly individualized, and what works for one person may not work for another [20,23].

In summary, PEM is a more specific symptom of fatigue, often described as a *crash* or *flare* that is a hallmark feature of GWI, ME/CFS and long COVID and is distinct from general fatigue. Effective management involves pacing and personalized specific symptom-based strategies to minimize its impact on daily life is urgently warranted.

### 2.2. Autonomic Dysfunction

Autonomic dysfunction (dysautonomia) results from damage to nerves of the autonomic nervous system, which controls automatic body functions. As noted in the Introduction, autonomic dysfunction has been increasingly recognized as a shared clinical feature across GWI, FM, ME/CFS, and long COVID [24,25,26].

While each of these disorders has a distinct origin, they converge in exhibiting abnormalities in autonomic nervous system regulation, particularly involving sympathetic and parasympathetic nervous system imbalance, orthostatic intolerance, heart rate variability, and blood pressure dysregulation [24,26]. In Gulf War veterans, autonomic dysfunction (particularly affecting cholinergic pathways) manifesting as dizziness, postural orthostatic tachycardia syndrome (POTS) and gastrointestinal dysfunction has also been reported [27,28,29].

Dysautonomia is a common feature of ME/CFS, particularly with regard to orthostatic intolerance, gastrointestinal problems, and body thermoregulation [30,31]. Patients with fibromyalgia also experience dysautonomia related symptoms of orthostatic intolerance, gastrointestinal dysfunction and problems with temperature regulation [32,33,34]. In long COVID patients, dysautonomia related symptoms including orthostatic intolerance, breathlessness and palpitations have been described, resulting from virus induced disruption of the autonomic nervous system [35,36,37]. In GWI and ME/CFS, autonomic dysfunction contributes to orthostatic hypotension syndrome and postural orthostatic tachycardia syndrome (POTS) [38]. It should be noted that in addition to autonomic dysfunction, symptoms such as palpitations, breathlessness, arrhythmia, and/or thermoregulation alterations (i.e., cold extremities) could also result from mitochondrial dysfunction in these disorders [39].

### 2.3. Cognitive Impairment

Cognitive impairment is a common feature of GWI, FM, ME/CFS and long COVID. Gulf War Illness is associated with cognitive impairments; including impaired memory and executive functioning (e.g., decision making, problem solving). Studies on GW veterans have demonstrated cognitive and mood impairments are linked to various adverse changes in neurons and neuroglial cells resulting in neuroinflammation [40,41,42,43,44,45].

There is also evidence for changes in brain structure and function associated with cognitive dysfunction in ME/CFS. For example, imaging studies in ME/CFS patients have found reduced gray matter density in certain brain regions associated with pain processing and cognitive function [46]. There is also evidence suggesting changes in brain activity in ME/CFS patients (and also long COVID), particularly in regions associated with memory, pain processing, and autonomic control [6]. In ME/CFS, elevated levels of pro-inflammatory cytokines/chemokines and growth factors have also been reported in patient blood samples, and there is evidence for neuroinflammation affecting the brain and spinal cord [47,48].

Fibromyalgia is often associated with cognitive impairment, commonly referred to as *fibro fog* [49]. This can manifest as difficulties with memory, concentration, attention, and overall mental clarity. While the exact cause of *fibro fog* is not fully understood, it is thought to be related to the chronic pain and fatigue associated with the disorder. In FM, evidence for elevated levels of pro-inflammatory cytokines/chemokines and growth factors, together with neuroinflammation, has also been reported [50]. The cognitive impairment may also be linked to changes in neurotransmitter and neural pathways involved in pain processing and cognitive function. While there is no cure for *fibro fog*, various strategies can help to manage symptoms such as some medications, cognitive behavioral therapy (CBT), lifestyle modifications, pacing activities, and support groups [51,52]. 

Cognitive impairment is a significant symptom of long COVID, with more than 50% of patients having cognitive slowing [53]. This cognitive impairment may result from neuroinflammation triggered by the immune response to the virus, or damage to blood vessels induced by the virus affecting blood flow to the brain [54]. Elevated markers of myeloid inflammation and complement activation have been identified in blood samples from long COVID patients [55].

### 2.4. Unrefreshing Sleep

Unrefreshing sleep is a prominent symptom of patients with GWI, FM, ME/CFS and long COVID. The exact cause of unrefreshing sleep is not fully understood in these disorders, although research suggests it may be related to disruptions in sleep architecture or alterations in autonomic nervous system function during sleep [56,57,58]. 

A significant percentage of GW veterans report experiencing unrefreshing sleep, along with other sleep-related issues such as difficulty falling asleep or staying asleep. Unrefreshing sleep is often accompanied by other symptoms that characterize GWI, including persistent fatigue, widespread pain, and cognitive difficulties [56]. This lack of restorative sleep can exacerbate other illness symptoms, affecting veterans’ overall health and well-being. Researchers are actively investigating the underlying mechanisms of GWI, including the role of sleep disturbance in its manifestation and progression. While there is no single cure for GWI, treatments like cognitive behavioral therapy for insomnia (CBT-I) have shown promise in improving sleep quality and reducing GWI symptoms [59].

FM is strongly associated with unrefreshing sleep, meaning that even after a full night’s sleep; individuals with FM often wake up feeling tired and not rested [57]. This is a core symptom, and it is often linked to other FM symptoms like disabling fatigue, chronic pain, and cognitive problems. People with FM often experience a disrupted sleep cycle/architecture, with more time spent in lighter stages of sleep (like stage 1) and less time in deep, restorative sleep (like slow-wave sleep) [60].

Several factors contribute to the sleep disturbances associated with FM. Individuals with FM tend to have more awakenings or arousals during the night, further disrupting the sleep cycle. Chronic pain can disrupt sleep, and poor sleep can exacerbate pain. This creates a vicious cycle where sleep problems worsen pain, and pain makes it harder to sleep [61]. Some research suggests that autonomic nervous system dysfunction might contribute to sleep problems in FM, leading to heightened arousal and difficulty relaxing into sleep [62]. By addressing sleep disturbances and chronic pain, individuals with FM can potentially improve their overall sleep quality and experience a greater sense of well-being.

Addressing sleep disturbance is crucial for managing FM. Treatment may involve improving sleep hygiene, establishing a regular sleep schedule, creating a relaxing bedtime routine, and optimizing the sleep environment, all of which are important [63]. Cognitive behavioral therapy for insomnia (CBT-I) helps individuals identify and change negative thoughts and behavior related to sleep [64]. In addition, sleep hygiene and conservative management is most appropriate for FM patients, along with ruling out or treating other confounding conditions such as obstructive sleep apnea, pain, anxiety, and depression [65].

Unrefreshing sleep is also a hallmark symptom of ME/CFS and long COVID [58,66]. This lack of restorative sleep significantly contributes to the overall fatigue and chronic pain and other common symptoms experienced by individuals with ME/CFS and long COVID. Many patients report not feeling rested; people with these disorders often wake up feeling just as tired, or even more tired, than when they went to bed. This persistent lack of restorative sleep contributes to the debilitating fatigue and widespread pain and other core symptoms associated with illness, affecting physical and mental well-being [67]. While objective sleep studies (like polysomnography) may not always show significant differences between patients with ME/CFS and long COVID and healthy individuals, the subjective experience of unrefreshing sleep is a major complaint and diagnostic criterion in these disorders [68,69].

In summary, unrefreshing sleep is a significant and troublesome symptom in these low-energy associated disorders, contributing to the overall burden of the illness and influencing the quality of life for those affected.

### 2.5. Muscle and Joint Pain

Muscle and joint pain are cardinal symptoms common to GWI, FM, ME/CFS, and long COVID, although the presentation and pathophysiological mechanisms may differ from each other [3,70,71]. Whilst all these disorders can cause widespread pain, they also have unique characteristics and specific diagnostic case criteria.

GWI is characterized by a range of symptoms including muscle and joint pain resulting from exposure to environmental mitochondrial toxicants during the Gulf War [70]. Pain is common and widespread in GWI patients, and their health-related quality of life is poor [72].

FM is often described as a persistent painful aching sensation, and it may be more intense in specific areas or fluctuate in frequency and severity. Pain is often felt in muscles, ligaments, and tendons, particularly at the points where they attach to bones. There is no single test for FM, and diagnosis is often based on symptom assessment [71]. Muscle and joint pain are also significant common features of ME/CFS and long COVID [3]. Although widely variable, fatigue, muscle and joint pain, and brain fog are frequently reported. Recent research indicates that a significant percentage of individuals with long COVID meet also the case criteria for ME/CFS and FM [3].

While all these disorders involve muscle and joint pain, the location and intensity can vary among sufferers. FM is characterized by widespread pain with specific tender points, while GWI, ME/CFS and long COVID pain may be more generalized or specific to certain areas (i.e., tender points in GWI patients may worsen after exertion) [6].

**Figure 1:**
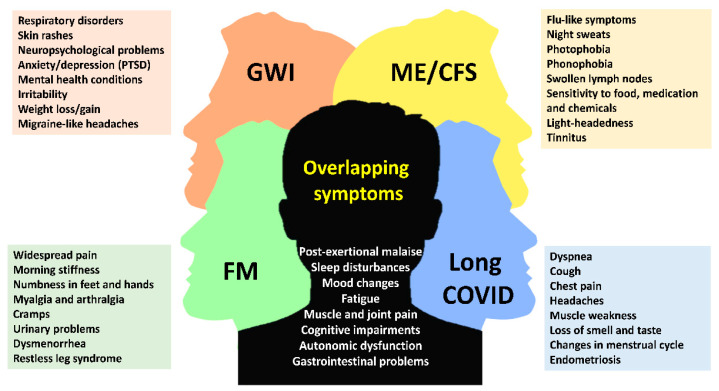
Schematic representation shows the most common symptoms among low-energy associated disorders. The figure highlights shared clinical features as well as those that are unique to each disorder. GWI, Gulf War Illness; FM, fibromyalgia; ME/CFS, myalgic encephalomyelitis/chronic fatigue syndrome; long COVID, post-COVID syndrome; PTSD, post-traumatic stress disorder.

In summary, muscle and joint pain are prominent features of GWI, FM, ME/CFS, and long COVID. While these disorders can be distinguished by their unique clinical characteristics and diagnostic case criteria, they can also overlap, particularly in their clinical presentation as shown in Figure 1.

## 3. Understanding the Biological Mechanisms Shared Between These Disorders

### 3.1. Mitochondrial Dysfunction, Oxidative Stress and Inflammation

Mitochondrial dysfunction, which affects cellular energy production, has been increasingly implicated in GWI, FM, ME/CFS, and long COVID [14]. There is some evidence suggesting that mitochondrial dysfunction may contribute to the profound fatigue and muscle weakness seen in these disorders. Previous studies have reported abnormalities in mitochondrial structure, impaired mitochondrial respiration, decreased ATP production, and abnormal mitochondrial DNA levels, as described below [73].

In GWI, exposure to chemical agents such as organophosphates or pyridostigmine bromide caused mitochondrial dysfunction in GW veterans. Chen et al. have identified damage to mitochondrial DNA [74], and Koslik et al. [75] and Golomb et al. [76,77,78] have reported impaired mitochondrial bioenergetic function in GWI veterans. In the latter studies, mitochondrial respiratory chain function was assessed in muscle biopsies from GWI patients, or via ^31^P-NMR spectroscopy in GWI patients; GWI severity was shown to be related to mitochondrial haplogroups. Elevated levels of oxidative stress biomarkers in serum from Gulf War veterans have been reported; however, there is limited evidence for increased oxidative stress in GWI [45]. In animal models of GWI, biomarkers of oxidative stress have been reported to be increased, both at the systemic level, and particularly in the hippocampus, a region of the brain important for memory and cognitive function [14,79]. GWI is associated with a persistent inflammatory state, particularly in the central nervous system; elevated levels of inflammatory markers (C-reactive protein, interleukin-6) have been identified in blood samples from Gulf War veterans [41]. It is of note that increased levels of inflammation biomarkers may represent a protective adaptation in GWI.

In FM, impaired oxidative phosphorylation, and reduced mitochondrial ATP production have also been reported, with the degree of mitochondrial dysfunction correlating with the severity of FM symptoms [80]. In FM, several studies have found evidence of increased oxidative stress in patient blood samples, including elevated levels of lipid peroxidation and decreased levels of antioxidants, including CoQ10, superoxide dismutase and catalase [14,81]. Evidence for elevated levels of pro-inflammatory cytokines/chemokines and growth factors, together with neuroinflammation, have been reported in FM [50].

Mitochondrial dysfunction in ME/CFS results in impaired ATP production and reduced mitochondrial bioenergetic reserve capacity, correlating with illness severity, which could explain the post-exertional malaise experienced by these patients [73]. Muscle biopsies have confirmed mitochondrial degeneration, deletions in mitochondrial DNA, and reduced mitochondrial activity in ME/CFS [82,83,84]. In ME/CFS, increased plasma levels of oxidative stress biomarkers have also been identified [14]. Elevated levels of pro-inflammatory cytokines/chemokines and growth factors have been reported in blood from ME/CFS patients, and there is evidence for neuroinflammation affecting the brain and spinal cord [47,48].

In long COVID, research has identified evidence of mitochondrial dysfunction in the blood immune cells of these patients. One of the hallmarks of long COVID is post-exertional malaise, which can be directly linked to mitochondrial dysfunction and compromised ATP production [85]. Evidence for structural and metabolic abnormalities in mitochondria from long COVID patients has been recently documented by Szögi et al. [86]. Increased levels of oxidative stress biomarkers and reduced antioxidant levels have been reported in long COVID patients [87]. In long COVID, elevated markers of myeloid inflammation and complement activation have also been identified [55].

### 3.2. Immune Dysregulation

Gulf War Illness, ME/CFS, and long COVID all show signs of immune system dysfunction, triggered by environmental toxins and/or viral persistence. Inflammation or altered immune responses, including elevated levels of pro-inflammatory cytokines/chemokines and growth factors, have been reported in these disorders [38,88]. Fibromyalgia may not show overt immune dysfunction, but is thought to involve central sensitization, where the brain becomes hypersensitive to centralized nociceptive and nociplastic pain signals [89].

Immune dysfunction in GWI is linked to exposure of neurotoxins, and other environmental stressors during military service; elevated levels of antibodies targeted against infectious agents found in veterans with GWI most likely results from aberrant immune function are linked with mitochondrial dysfunction [4]. Elevated plasma levels of pro-inflammatory cytokines, including interferon-gamma, interleukin (IL)-2, IL-6 and tumor necrosis factor-alpha (TNF-α), together with elevated levels of C-reactive protein have been found in Gulf War veterans. Elevated plasma levels of T- and B-cells, and reduced levels of natural killer (NK) cells, have also been reported in veterans with GWI [90,91,92]. Long COVID is associated with persistent immune cell activation, autoimmune antibody production and immune exhaustion/senescence [93,94,95].

ME/CFS can also be triggered by viral infection persistence (e.g., Epstein-Barr virus, cytomegalovirus, herpesvirus, etc.). In ME/CFS, elevated blood levels of pro-inflammatory cytokines/chemokines and growth factors, together with reduced functional ability of NK cells and B- or T-cell abnormalities have been described. In particular there is evidence that NK cells and cluster of differentiation CD8+ T cells in ME/CFS may exhibit signs of exhaustion, potentially limiting their ability to effectively fight persistent infection [96,97].

FM is sometimes thought to be triggered by infections or physical/emotional trauma, although this is less clear [98,99]. There is evidence for immune system dysfunction in FM, with altered levels of cytokines/chemokines and growth factors and other inflammatory biomarkers in the brain and spinal fluid indicating neuroinflammation may play a potential role [100,101]. A study by Goebel et al. found that transfer of antibodies present in the blood of fibromyalgia patients could induce fibromyalgia symptoms in mice, suggesting an autoimmune component [102].

### 3.3. Neuroendocrine Abnormalities

Gulf War Illness, FM, ME/CFS or long COVID are associated with hypothalamic–pituitary–adrenal (HPA) axis neuroendocrine abnormalities, characterized by an attenuated stress response and reduced HPA axis activity, potentially leading to lower cortisol levels and enhanced sensitivity to glucocorticoid feedback [103,104,105]. In Gulf War veterans, alterations in the HPA axis (specifically suppression of adrenocorticotropic hormone (ACTH) and cortisol) has been linked to environmental exposures, including chemical warfare agents, pesticides, and other environmental stressors, possibly contributing to long-term GWI symptoms such as disabling fatigue, post-exertional malaise, dysautonomia, sleep disturbances, and cognitive impairments [106].

Hypothalamic–pituitary–adrenal axis neuroendocrine dysregulation, characterized by low cortisol levels (hypocortisolism) and altered cortisol rhythms is particularly evident in individuals with ME/CFS, where a dysregulated stress response can lead to unexplainable fatigue, post-exertional malaise, autonomic dysfunction, and sleep disturbances, among other core symptoms [107]. There is also some evidence of altered hormone levels in FM and long COVID, although this is less well established [108,109].

However, the exact cause and effect association and heterogeneity of these findings are still areas of ongoing research. More research is needed to fully understand the role of HPA axis neuroendocrine alterations, their relationship to neuroimmune function, and their potential as surrogate biomarkers or therapeutic targets in these disorders.

### 3.4. Dysregulated Gut–Brain Axis

Recent research suggests a significant link between GWI and the gut–brain axis. Specifically, GWI is associated with gastrointestinal problems and neurological symptoms, and studies indicate that alterations in the gut microbiome (dysbiosis and impaired intestinal permeability) may play a crucial role in the development and persistence of symptoms [110,111]. Several studies have shown that GW veterans have distinct differences in their gut microbiome composition compared to matched healthy individuals [112]. A significant portion of GW veterans experience gastrointestinal problems, including abdominal pain, diarrhea and irritable bowel syndrome (IBS), which are closely linked to illness [113].

GWI also involves neurological symptoms including fatigue, cognitive deficits, and mood changes, suggesting a potential involvement of the gut–brain axis in these manifestations. The gut–brain axis is believed to be disrupted in GWI through various mechanisms, including changes in the gut microbiome, increased intestinal permeability (leaky gut), and altered enteric nervous system (ENS) function [114]. Understanding the role of the gut–brain axis in GWI opens avenues for developing novel potential therapeutic strategies, such as targeting the gut microbiome or modulating the ENS [115]. It is also of note that since Gulf War veterans have higher rates of infection, they may have received more antibiotics, which in turn will result in an altered gut microbiome, irrespective of whether this plays a causal role in gastrointestinal symptoms in GWI.

Studies using a mouse model of GWI have shown that the compound andrographolide can restore gut microbiome balance, improve gut barrier function, and reduce neuroinflammation, potentially alleviating GWI symptoms [116]. Exposure to pyridostigmine bromide, a medication used during the Gulf War, has been linked to gut dysbiosis and gastrointestinal neuroimmune disruption in animal models [117]. 

In summary, the gut–brain axis is a critical area of research in understanding the complexities of GWI, with evidence suggesting that gut microbiome alterations, gastrointestinal dysfunction, and neurological symptoms are interconnected. Further research into the gut–brain axis in GWI could lead to more effective treatments and management strategies for GW veterans.

Fibromyalgia is increasingly linked to disruptions in the gut–brain axis, often involving dysbiosis and altered gut permeability, contributing to the wide range of FM symptoms [118,119]. The gut–brain axis is a bidirectional communication system that connects the central nervous system with the enteric nervous system [120]. This communication occurs through various pathways, including the vagus nerve that plays a crucial role in relaying signals between the gut and the brain. The HPA axis, involved in stress response, can be impacted by gut health; the gut is a major site of immune activity, and immune cells can influence both gut and brain function [121]. 

The gut microbiome influences the production and regulation of neurotransmitters (such as imbalances of serotonin and dopamine), which play a potential role in mood, anxiety/depression, pain perception, and cognitive function. In addition, several studies have described how dysregulation of the gut–brain axis can contribute to central sensitization, a phenomenon where the nervous system becomes overly sensitive to pain signals [111,114,121].

Myalgic encephalomyelitis/chronic fatigue syndrome and long COVID exhibit disruptions in the gut–brain axis, which involve alterations in gut microbiota composition, immune responses, and neurotransmitter signaling, potentially contributing to the diverse and debilitating symptoms seen in both disorders [122,123]. Both disorders are associated with imbalances in the gut microbiome, including shifts in bacterial populations and reduced microbial diversity. For example, in ME/CFS, specific bacterial groups such as *Alistipes* may be increased, while *Faecalibacterium* is decreased [124,125,126]. In long COVID, prolonged fecal shedding of SARS-CoV-2 and associated inflammation could lead to dysbiosis [127,128,129,130,131].

Understanding the role of the gut–brain axis in these disorders opens up new avenues for treatment with probiotics and prebiotics that may help restore gut microbiome balance; dietary interventions to reduce inflammation and promote a healthy gut environment could also be beneficial. Vagus nerve stimulation, which can be achieved through various methods, may help modulate gut–brain communication. Further research into the intricate interplay between the gut and the brain is crucial for developing more effective and targeted therapies in these disorders [115,122,132]. 

### 3.5. Apoptosis and Ferroptosis

Apoptosis is a process of programmed cell death, which can occur after a number of triggering events, including mitochondrial dysfunction and oxidative stress [133]. Ferroptosis is a type of iron-dependent programmed cell death (distinct from apoptosis) characterized by the accumulation of free radical induced lipid peroxidation products, with key regulators including GPX4 and the SLC7A11/GSH system and FTH1, which counteract lipid peroxidation [134]. There is evidence that apoptosis and/or ferroptosis contribute to the pathogenesis of GWI, ME/CFS, FM and long COVID.

In GWI, serum from patients has been reported to increase apoptosis in cultured neuroblastoma cells, suggesting that apoptosis potentially contributes to the neurocognitive and mood dysfunction experienced by some veterans [135]. Such data related to apoptosis in GWI research have opened avenues for potential therapeutic interventions. For example, Tsilibary et al. investigated the role of the anthrax vaccine given to Gulf War veterans in inducing apoptosis in cultured neural cells, and the use of IgG isotype anti-protective antigen protein antibodies in neutralizing the adverse effects of serum on neural cultures, including reducing apoptosis [136]. This research suggests that immunotherapy targeting specific antigens or pathways involved in GWI-related apoptosis could be a viable approach for treating the illness.

In fibromyalgia, there is some evidence that increased apoptosis in nerve cells, particularly dorsal root ganglia, may contribute to nerve damage and altered pain processing, resulting in the pain and hypersensitivity associated with this disorder. Apoptosis-related genes are reportedly upregulated in dorsal root ganglia in models of neuropathic pain, suggesting a potential link to chronic pain disorders like FM [137]. Increased apoptosis markers and decreased cell viability have been observed in animal models of FM, factors that can be reduced following selenium administration [138]. Transient receptor potential (TRP) channels (like TRPM2 and TRPV1) play a role in calcium influx into nerve terminals, which can trigger apoptosis. Selenium treatment in animal models noted above reduces hyperalgesia, oxidative stress levels, and apoptosis by modulating these channels [138]. Elevated levels of anti-annexin antibodies, which are associated with apoptosis, have been reported in some FM patients, particularly those with Sjögren’s syndrome [139]. Similarly, there is evidence that ferroptosis, particularly in skeletal muscles, may play a role in the widespread pain and other symptoms associated with FM [140]. Specifically, studies have shown that magnesium hexacyanoferrate nanocatalysts, which act as ferroptosis inhibitors, can alleviate FM symptoms in mouse models by reducing oxidative stress and mitochondrial dysfunction [141].

Patients with ME/CFS have an increased rate of apoptosis in their neutrophils compared to healthy individuals; neutrophils are thus eliminated at a faster than normal rate, which may impair the immune system’s ability to respond effectively to pathogenic agents [142]. Cezar et al. found CD4+ T cells from participants who later presented long COVID were more apoptotic in culture than those of sequelae-free participants at month 1, suggesting that CD4+ T cell death during the acute phase of SARS-CoV-2 infection might pave the way for long COVID [143]. In particular with regard to the potential of ferroptosis and oxidative stress pathways in the pathogenesis of these disorders, Biamonte et al. reported a correlation between circulating iron status and hematological index indicators during the COVID-19 progression [144], and a low baseline level of a ferroptosis marker (SLC7A11) may indicate an increased risk for long COVID-19, and associated cardiovascular and neurological dysfunction [145,146,147].

### 3.6. Telomere Shortening

Telomeres are repetitive DNA sequences at the ends of chromosomes that protect the chromosomes from damage and degradation. During DNA replication DNA polymerase, the enzyme that copies DNA, cannot fully replicate the very ends of the chromosomes, leading to a small loss of telomere length with each cell division; this shortening of the telomeres eventually leads to cellular senescence and apoptosis [148]. Whilst telomere shortening is a natural part of the aging process, a number of factors can accelerate this process, including lifestyle factors and exposure to toxins [149]. At the cellular level, telomere shortening is linked to mitochondrial dysfunction, oxidative stress, and inflammation [150,151,152]. Telomere shortening leads to premature cellular aging and increased risk of age-related diseases [153].

Elevated mitochondrial ROS levels directly damage telomeric DNA by oxidizing guanine bases, creating lesions such as 8-oxoguanine [150,151,152]. Telomeric 8-oxoguanine lesions can disrupt the binding of essential telomere-binding proteins (TRF1 and TRF2) as part of the shelterin complex, which are crucial for protecting telomere stability and maintenance [150,151,152].

There is evidence for telomere shortening in military veterans with GWI, FM, ME/CFS and long COVID. Military veterans are reported to have shorter telomeres compared to their civilian peers [154]. Gulf War veterans were exposed to a variety of environmental toxins, including pesticides, *pyridostigmine bromide*, and smoke from oil well fires [155]. Such exposures are linked to mitochondrial dysfunction, oxidative stress and inflammation [156], thereby accelerating telomere shortening. Veterans with GWI may therefore have shorter telomeres compared to their peers without the illness, although this requires confirmation. Such accelerated telomere shortening could be a contributing factor to the development of various health problems associated with GWI, including cardiovascular and neurodegenerative disorders [154,156].

There is evidence that individuals with ME/CFS experience a faster rate of telomere shortening compared to healthy individuals, indicating premature aging. A study by Rajeevan et al. found that the association between ME/CFS and shorter telomeres was largely moderated by females under 45 years old [157]. The observed telomere shortening in ME/CFS could be a marker of premature immunosenescence (premature aging of the immune system) and may contribute to the development of other age-related diseases.

Fibromyalgia is also associated with telomere shortening, with the degree of shortening correlating with the frequency/severity of pain [158]. When FM is accompanied by anxiety/depression, the effects on telomere length can be even more pronounced in these patients. These data suggest that chronic pain in FM may contribute to premature cellular aging, affecting overall health and potentially increasing the risk of age-related diseases [158].

Research suggests that acute SARS-CoV-2 infection can accelerate telomere shortening in blood cells, potentially contributing to the persistent symptoms associated with long COVID [159,160,161]. The telomere shortening observed in long COVID appears to be independent of the patient’s chronological age, suggesting a direct impact of the virus on telomere length [161]. Patients with shorter telomeres in the immune cells (leukocytes) have been linked to worse prognoses in COVID-19, and this shortening may be independent of chronological age [159].

**Figure 2:**
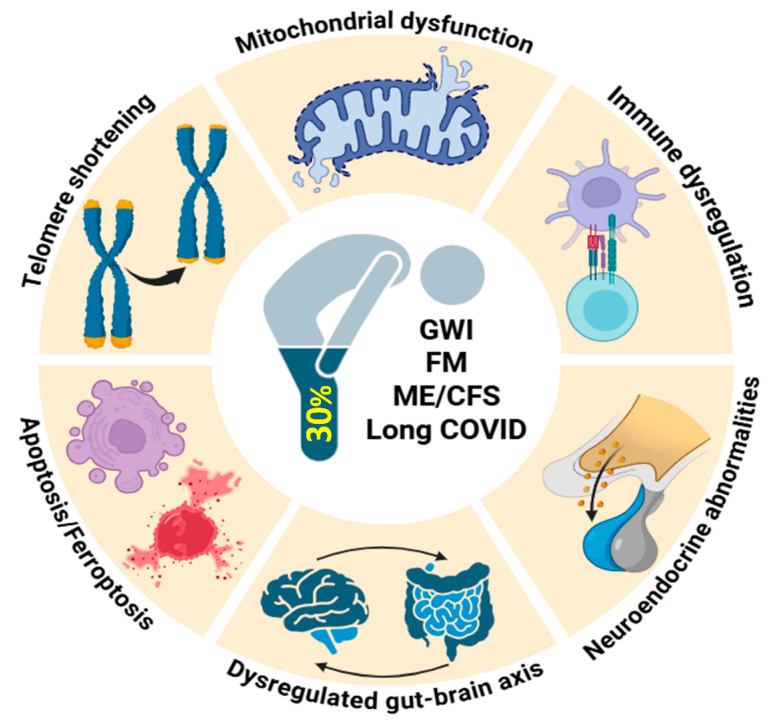
Potential shared pathophysiological mechanisms underlying low-energy associated disorders. The schematic overview highlights how pathologies with different origins can converge on common pathophysiological mechanisms, illustrating a complex interplay that leads to overlapping symptoms observed in these disorders. GWI, Gulf War Illness; FM, fibromyalgia; ME/CFS, myalgic encephalomyelitis/chronic fatigue syndrome; long COVID, post-COVID syndrome. Created with BioRender (BioRender software version 04. https://app.biorender.com; accessed on 4 August 2025).

In summary, Figure 2 displays shared biological abnormalities in the development of low-energy associated disorders.

## 4. Implications for Current and Promising Therapeutic Strategies

### 4.1. Current Therapeutic Approaches

There are no specific cures for GWI, FM, ME/CFS and long COVID but current treatments are focusing on improving quality of life through symptom management and non-pharmacological approaches like pacing (activity management), cognitive-behavioral therapy (CBT), graded exercise therapy (GET), relaxation, yoga, Tai-Chi, and acupuncture [65,162,163,164,165]. For symptoms like anxiety/depression, some antidepressants, including tricyclic and heterocyclic antidepressants, monoamine oxidase inhibitors (MAOIs) and selective serotonin reuptake inhibitors (SSRIs) may be used amongst disorders [65,162]. The treatments are individualized, with a focus on active management and coping strategies, and are often coordinated with health coaching and care management. Mindfulness-based therapies and CoQ10 supplementation have shown potential benefits for managing symptoms of sleep disturbances, chronic pain and fatigue in these chronic multisymptom disorders [65,162,163,164,165]. In summary, there are ongoing research efforts to better understand these complex disorders and develop new diagnostic criteria, potential biomarkers and targeted treatments.

### 4.2. Stem Cell Therapy

Stem cells can differentiate into various cell types, promoting tissue regeneration and potentially repairing damaged organs. Based on their ability to differentiate into other cell types, stem cells are classified as either pluripotent, multipotent, omnipotent, totipotent, oligopotent, or unipotent. Depending on where they originated from, stem cells may be classified as embryonic, adult, fetal, or induced pluripotent stem cells (iPSCs) [166]. Stem cell therapy involves transplanting healthy stem cells to replace damaged or diseased stem cells, or using them to stimulate the body’s own repair mechanisms, for example, by secreting growth factors or recruiting other cell types necessary for tissue repair [167].

Stem cells can transfer their healthy mitochondria to cells with damaged or dysfunctional mitochondria, thereby restoring cellular energy levels and reducing oxidative stress; this transfer can occur through various mechanisms, including tunneling nanotubes, gap junctions, and extracellular vesicles [168]. In addition to reducing oxidative stress by improving mitochondrial function, stem cells per se have antioxidant action, directly scavenging free radicals or stimulating production of antioxidant enzymes like superoxide dismutase, catalase, and glutathione peroxidase in other cells [169]. Stem cells can also engulf and degrade dysfunctional mitochondria from damaged cells, promoting cellular repair and proliferation; the engulfment of damaged mitochondria can trigger the production of cytoprotective enzymes like heme-oxygenase-1 (HO-1), which can further enhance the stem cell’s ability to rescue damaged cells [170]. Additionally, stem cells can release factors that stimulate mitochondrial biogenesis in recipient cells. Stem cells can also help to regulate the immune system, reducing inflammation via shifting the balance of immune cell activity from a pro-inflammatory to an antiinflammatory state [171].

Stem cell therapy is of relevance to the treatment of GWI, FM, ME/CFS, and long COVID, although research in each of these areas is still at an early stage. Mesenchymal stem cells may be particularly beneficial in treating patients with ME/CFS; improvements in sleep quality, levels of energy and pain, and cognitive function have been reported following stem cell therapy. Several randomized controlled clinical trials have reported the beneficial effects of stem cell therapy in patients with severe COVID-19 infection [172,173,174]. Mokhemer et al. reported symptomatic improvement in a rat model of FM following stem cell therapy [175]. Using a cell culture based system; Tsilibary et al., identified the potential of stem cell therapy to protect cells against toxic factors present in serum from veterans with GWI [19].

### 4.3. Coenzyme Q10 Supplementation

Supplementation with CoQ10 has been shown to be beneficial in GWI, FM, ME/CFS and long COVID, resulting from improved mitochondrial function, reductions in oxidative stress, inflammation and apoptosis/ferroptosis, and telomere shortening, as described below.

In GWI, supplementary CoQ10 improved physical function and self-reported health, as well as fatigue, pain and muscle strength [176]. Several studies have shown that CoQ10 supplementation can reduce fatigue and improve quality of life in individuals with ME/CFS. Castro-Marrero et al. reported supplementation with CoQ10 and nicotinamide adenine dinucleotide (NADH) improved fatigue in ME/CFS [177]. Supplementation with CoQ10 for symptoms such as fatigue, pain, and cognitive dysfunction in ME/CFS has been suggested in a consensus report from the European network on ME/CFS (EUROMENE consortium) [178].

Fibromyalgia patients have depleted CoQ10 levels in tissues (typically 40–50% of the normal level) [179]. Hence, supplementation with CoQ10 can help reduce chronic pain, fatigue, and improve overall quality of life in FM patients. A randomized, double blind, placebo-controlled study found that 300 mg/day of CoQ10 for 40 days significantly reduced chronic pain and fatigue in FM patients, with a corresponding improvement in mitochondrial energy production [179]. Supplemental CoQ10 may be particularly helpful in reducing pain and fatigue in those with FM who are also taking pregabalin [180].

Clinical studies supplementing CoQ10 in COVID-19 patients (and in patients with long COVID) have reported mixed outcomes. In a prospective observational study, 116 patients with long COVID were supplemented with 200 mg of CoQ10 and 200 mg of alpha-lipoic acid per day for 2 months versus 58 long COVID patients who received placebo; the fatigue severity assessed using self-reported questionnaire was substantially reduced in the treated patients compared to placebo [181]. However, a randomized controlled intervention study comprising 121 long COVID patients supplemented with 500 mg of CoQ10 per day for 6 weeks reported no significant benefit on illness symptoms [182].

There is evidence that supplementation with CoQ10 may help protect telomeres from damage caused by oxidative stress and inflammation. A study involving older adults with low selenium levels showed that supplementation with CoQ10 and selenium resulted in less telomere shortening compared to placebo. This study also found a correlation between longer telomeres and reduced cardiovascular mortality [183].

There is also evidence that supplementary CoQ10 could improve the efficiency of stem cell therapy when this procedure is applied in these disorders. A number of studies have demonstrated beneficial effects of CoQ10 on stem cell metabolism, of relevance to improving the efficiency of stem cell therapy when applied in the above disorders. Coenzyme Q10 has been shown to protect stem cells from damage caused by factors like hypoxia, oxidative stress, and aging [184].

For example, CoQ10 has been shown to protect bone marrow-derived mesenchymal stem cells (BMSCs) from H_2_O_2_-induced oxidative stress and cell death. It has also been shown promise in reducing oxidative stress and potentially enhancing the therapeutic efficiency of BMSC transplantation for spinal cord injury treatment [185,186]. Coenzyme Q10 can enhance the regenerative potential of stem cells by promoting cell differentiation, angiogenesis, and tissue repair. It can also help stem cells migrate to the site of injury and integrate into the damaged tissue. Coenzyme Q10 can be combined with stem cells in various ways, such as co-administration or loading stem cells with CoQ10. For example, CoQ10-loaded exosomes have shown promise in delivering CoQ10 to target cells and enhancing their therapeutic effects [187,188,189].

Coenzyme Q10 has shown promise in enhancing stem cell therapy for conditions like lung fibrosis, nerve injury, and intervertebral disk degeneration [190]. Maruo et al. described the activation of mitochondria in human mesenchymal stem cells using encapsulated CoQ10 [191]. Li et al. identified impaired mitochondrial function in mesenchymal stem cells of aged mice compared to young mice, and suggested that this area of metabolism as a potential therapeutic target (i.e., via CoQ10 supplementation) to enhance the regenerative function of these cells [192]. Studies to investigate the effect of CoQ10 in reducing stem cell senescence, improving the survival rate and activity of stem cells against toxic agents, and increasing the efficiency of transplanted stem cells have been summarized in Table 1.

It is important to note that individual responses to CoQ10 supplementation may vary, and further research is needed to fully understand its therapeutic implications for these disorders.

## 5. Conclusions and Future Perspectives

Although GWI, FM, ME/CFS and long COVID have distinct origins, it should be noted that a significant proportion of GWI veterans meet case criteria for ME/CFS and FM. This review explores the evidence that these disorders comprise a group of so-called low-energy associated disorders with overlapping common symptoms and underlying to shared pathophysiological mechanisms. In particular, evidence for the roles of mitochondrial dysfunction, oxidative stress, inflammation, apoptosis/ferroptosis, immune dysregulation, autonomic dysfunction, disrupted brain–gut–microbiome axis and telomere shortening as common features in the pathogenesis of these disorders has been identified.

In this review, we have explored the overlapping shared symptoms and pathological features that are common to these disorders. One might therefore question why the above have been classified as four distinct disorders, rather than a single disorder. Given that there are lack of specific biomarkers and accurate objective case criteria to differentiate these disorders; particularly stems from their inherent multifactorial nature, heterogeneity, and overlapping common symptoms and underlying biological pathomechanisms. All the above leads to subjective clinical diagnosis, difficulties in comparing research findings, a higher risk of patients receiving ineffective interventions, and an inability to identify specific patient subgroup for developing targeted therapies. While some progress has been made with biomarkers for these disorders, significant challenges remain in discovery, validation, stratification, and translating findings into reliable clinical tools due to complexities in underlying pathology and intra- and inter-patient sample variations.

Given the potential role of CoQ10 in promoting normal mitochondrial function, as an antioxidant, antiinflammatory and antiapoptotic/antiferroptotic agent, there is a rationale for the role of supplementary CoQ10 in the management of these disorders. The reported benefits of supplementary CoQ10 administration in reducing telomere shortening and improving the efficiency of stem cell transfer has also been identified as promising therapeutic strategies in these disorders.

Although previously published reports have explored shared common symptoms and biological abnormalities in ME/CFS and long COVID, to our knowledge this is the first study to summarize overlapping common symptoms and specific pathophysiological mechanisms in GWI, FM, ME/CFS and long COVID, and how these might be addressed via CoQ10 supplementation and stem cell therapy.

While promising, further research and rigorous large-scale randomized controlled trials are needed to fully elucidate the underlying biological pathomechanisms by which CoQ10 can mitigate the common symptoms, and to establish efficacy and dosing protocols in these disorders. Understanding these issues, connections based on a personalized medicine approach may provide new targets for therapeutic interventions and improve outcomes for individuals with these complex disorders.

## Figures and Tables

**Table 1:** Summary of studies supplementing Coenzyme Q10 in stem cell models.

Study Refs.	Models	Outcomes
Park et al. (2012) [193]	Neural stem cells (rat)	Improved cell viability and intracellular signaling proteins during hypoxia-reperfusion
Choi et al. (2013) [194]	Neural stem cells (mouse)	CoQ10 restored amyloid beta-inhibited proliferation by activating the PI3K pathway
Zhang et al. (2015) [184]	Mesenchymal stem cells (rat)	Inhibition of oxidative stress and cell aging induced by D-galactose
Lee et al. (2021) [195]	Ovarian stem cells (mouse)	Improved stem cell function in vinylcyclohexene-diepoxide induced model of ovarian failure
Velichkovska et al. (2019) [196]	Neural progenitor cells (mouse)	Mitochondrial dysfunction induced by anti-retroviral drugs (tenofovir and emtricitabine) improved
Liu et al. (2022) [197]	Airway basal stem cells (human)	Reduced oxidative stress induced by hydrogen peroxide; improved efficiency of transplanted cells in bleomycin-induced model of pulmonary fibrosis
Sun et al. (2023) [190]	Mesenchymal stem cells (rat)	Improved efficiency of transplanted cells in rat model of intervertebral disk degeneration
Hernández-Perez et al. (2022) [198]	Mesenchymal stem cells (human)	Reduced oxidative stress, improved cell viability and proliferation following exposure to MPP+
Zheng et al. (2023) [199]	Umbilical cord mesenchymal stem cells (human)	Reduced oxidative stress induced by hydrogen peroxide, cell senescence reduced and proliferation capacity improved

## Data Availability

Not applicable.

## Abbreviations

The following abbreviations are used in this manuscript:

ANS	Autonomic nervous system
ATP	Adenosine triphosphate
BMSC	Bone marrow-derived mesenchymal stem cells
CBT-I	Cognitive behavior therapy-insomnia
CDC	Centers for Disease Control and Prevention
CoQ10	Coenzyme Q10
ENS	Enteric nervous system
FM	Fibromyalgia
FDA	Food and Drug Administration
FTH1	Ferritin heavy chain 1
GET	Graded exercise therapy
GPX4	Glutathione peroxidase 4
GWI	Gulf War Illness
HRV	Heart rate variability
HPA	Hypothalamic–pituitary–adrenal axis
HO-1	Heme-oxygenase-1
IBS	Irritable bowel syndrome
IL	Interleukin
MAOI	Monoamine oxidase inhibitors
ME/CFS	Myalgic encephalomyelitis/chronic fatigue syndrome
MPP+	1-methyl-4-phenylpyridinium
NADH	Nicotinamide adenine dinucleotide
NK	Natural killer cells
PEM	Post-exertional malaise
PESE	Post-exertional symptom exacerbation
PI3K	Phosphatidylinositol-3-kinase
POTS	Postural orthostatic tachycardia syndrome
iPSC	Induced-pluripotent stem cells
RCT	Randomized controlled trial
ROS	Reactive oxygen species
SARS-CoV-2	Severe acute respiratory syndrome coronavirus 2
SLC7A11	Solute carrier family 7 member 11
SSRI	Selective serotonin reuptake inhibitors
TRPV1	Transient receptor potential vanilloid 1
TRPM2	Transient receptor potential melastatin 2
TRF1	Telomeric repeat-binding factor 1
TRF2	Telomeric repeat-binding factor 2
